# Does the enterolactone (ENL) affect fatty acid transporters and lipid metabolism in liver?

**DOI:** 10.1186/s12986-017-0223-1

**Published:** 2017-11-13

**Authors:** Krzysztof Drygalski, Klaudia Berk, Tomasz Charytoniuk, Nicoletta Iłowska, Bartłomiej Łukaszuk, Adrian Chabowski, Karolina Konstantynowicz-Nowicka

**Affiliations:** Department of Physiology, Medical University of Bialystok, Białystok, Poland

## Abstract

**Background:**

NAFLD as a result of inappropriate diet and obesity, may progress to sever conditions such as: type 2 diabetes mellitus or steatohepatitis, and has recently become a prevalent topic of numerous investigations. Due to its dangerous aftermaths, finding new substances, such as polyphenols and their derivatives, which might reduce liver steatosis is the main target of research into NAFLD treatment. Hence, the aim of the present study was to evaluate the effect(s) of enterolactone (ENL), a metabolite of secoisolariciresinol (SECO), on lipid metabolism together with changes in the expression of fatty acid transporters in fatty liver.

**Methods:**

The experiments were conducted on HepG2 cells incubated with either ENL and/or palmitic acid during 16 h exposure. The expression of selected fatty acid transport proteins: FATP2, FATP5, CD36, FABPpm, ABCA1, MTP, ACBP and L-FABP, as well as the proteins directly involved in lipogenesis (FAS), oxidation pathway (CPT 1), and lipid metabolism (PPARα, LXR, SREBP1c, pAMPK) was estimated by Western Blot. Intra and extracellular lipid contents were assessed by Gas-Liquid Chromatography. The data was analyzed with two-way analysis of variance (ANOVA), and results were considered to be statistically significant at *p* ≤ 0.05.

**Results:**

ENL stimulated extracellular efflux of free fatty acids (FFA) and triacylglicerols (TAG) to the medium, while, it had no influence on FATP-family mediated intracellular fatty acid uptake. Moreover, ENL decreased the expression of CPT 1, pAMPK, PPARα, increased SREBP1c and had no effect on LXR, and FAS content.

**Conclusions:**

The findings of our study demonstrate that ENL had opposite effect on liver steatosis in comparison with other polyphenols what suggests that it may be an inactive metabolite. ENL did not affect significantly the intracellular accumulation of FFA, DAG and TAG, yet it promoted their extracellular efflux. Furthermore, it inhibited ß-oxydation and intracellular lipid metabolism what may contribute to the progression of NAFLD.

## Background

Non-alcoholic fatty liver disease (NAFLD) is one of the most common, diet related liver diseases appearing especially within societies of developed countries. It is thought that nowadays this pathology may occur in up to 33% of the general population [1, 2]. However, the risk of NAFLD may be underestimated due to the still increasing problem of obesity in many western countries, especially among youths [3]. That is why, obesity with accompanying hyperlipidemia, excluding increased alcohol consumption, are the main pathogenetic factors leading to liver steatosis [4]. High fat and high glucose diets result in an elevated free fatty acids (FFA) concentration in plasma, which leads to lipid accumulation in peripheral tissues, including hepatocytes [5]. In NAFLD, the liver accumulates mainly triacylglycerols (TAG), what might be seen in histopathological picture of thick needle biopsy (TNB) as various size cytoplasmatic lipid droplets. Transmembrane FFA uptake is mainly due to the presence of plasma membrane transporters such as: fatty acid translocase (FAT/CD 36), fatty acid transport proteins (FATP2, FATP5) and fatty acid binding protein (FABPpm) [6, 7]. Moreover, intracellular translocation of lipids highly depends on cytosolic liver-type fatty acid binding protein (L-FABP) and acyl binding protein (ACBP) which provide high enough concentration gradient for FA diffusion. An excessive intracellular FA flux into hepatocytes is esterified to triacylglycerols (TAG) and/or diacylglycerols (DAG) which may disturb cellular lipid metabolism, leading to the development of steatotic changes [6–8]. Furthermore, long lasting steatosis causes inflammation and progression to non-alcoholic steatohepatitis (NASH) which is a primary risk factor for cirrhosis and hepatocellular carcinoma [9]. Taking into consideration all the above-mentioned aftermath, it is clear that NAFLD is the first step in the development of many diseases caused by a disruption of cell metabolic pathways. As it was shown previously, many polyphenols or phytoestrogens are investigated nowadays as a potential medicine for NAFLD treatment and some of them, such as resveratrol, have reached the level of clinical trials, providing promising outcomes [10]. Based on actual knowledge we have chosen enterolactone (ENL), a phytoestrogen with proven anti-neoplastic and anti-diabetic properties for our study [11, 12]. ENL is a natural lignin composed of two phenol rings connected by one lactone ring. It can be found in many plants’ seeds such as flaxseed or sesame seed [11, 12]. What is more, ENL might also be synthesized by gut microbiota as a decay product of secoisolariciresinol (SECO) metabolism [13, 14]. Interestingly, the concentration of SECO in patients’ plasma is positively correlated with higher plasma HDL and decreased TAG concentration, which information was important in choosing ENL to our research [15]. However, ENL has not been examined before in the context of NAFLD development in connection with fatty acid transporters activity and lipid overload related impairment of the lipid metabolism [16, 15].

## Methods

### Cell culture

The experiments were conducted on HepG2/C3A an immortalized human liver cell line, obtained from ATCC (American Type Culture Collection). The cells were maintained on a standard growth medium (DMEM- Dulbecco Modified Eagle Medium) supplemented with 10% fetal bovine serum (FBS) and 1% penicillin/streptomycin for 5 days at 37 °C in a humidified atmosphere containing 5% of CO_2_ until they reached 70% confluency. During this period the cells were rinsed with PBS, and the medium was replaced every 48 h. After 5 days the cells were transferred to 6 well plates and cultured in the growth medium to achieve 90% of confluence. The morphology and viability of the HepG2 cells were assessed in Bürker chamber using Trypan blue staining.

### PA and/or ENL incubation

Briefly, palmitic acid before administration to the cells was dissolved in a solution of ethanol and 1 M NaOH, heated to 70 °C, mixed with fatty acid-free bovine serum albumin (2% BSA) and diluted in DMEM [17]. Three hours before performing the experiments cells were serum-starved and next incubated with enterolactone (50 μM) alone or combined with palmitic acid (0.5 mM) for 16 h. After this time, HepG2 cells were homogenized in ice-cold radioimmunoprecipitation assay (RIPA) lysis buffer containing protease inhibitors, ultrasonicated and together with postincubation media samples were taken, and frozen in liquid nitrogen. Protein concentration was determined with bicinchonic acid method using BSA as a protein standard.

### Immunoblotting analyses

Routine Western Blotting procedures were used to detect the total expression of fatty acid transport proteins: FATP2, FATP5 (Santa Cruz Biotechnology, USA), FAT/CD36, FABPpm (Abcam, UK), ABCA1 (Thermo Scientific, USA), MTP (Santa Cruz Biotechnology, USA), ACBP and L-FABP (Abcam, UK) as well as the proteins directly involved in lipogenesis (FAS; Cell Signaling, USA), oxidation pathway (CPT 1; Santa Cruz Biotechnology, USA) and lipid metabolism (PPARα, LXR, SREBP1c, pAMPK; Cell Signaling, USA) as previously described in details by Konstantynowicz-Nowicka et al. [17]. All the antibodies used in our procedures were monoclonal except for FATP2 and FAT/CD36. Cell lysates were separated by 10% sodium dodecyl sulfate-polyacrylamide gel electrophoresis (SDS-PAGE) and transferred to nitrocellulose membranes. After blocking with 5% nonfat dry milk, the membranes were immunoblotted with primary antibodies of interest and incubated with secondary antibodies labeled with horseradish peroxidase (HRP). Obtained protein bands were quantified densitometrically using a ChemiDoc visualization system (Bio Rad, Warsaw, Poland). Equal protein loading was controlled by Ponceau S staining. The expression of all the proteins was standardized to the GAPDH (Santa Cruz Biotechnology, USA) expression and the control was set as 100%.

### Intra- and extracellular lipid concentration analyses

As described previously [7], lipids from HepG2 cells as well as postincubation media were extracted according to the Folch method [18] and separated by thin-layer chromatography (TLC) into fractions of the free fatty acids (FFA), diacylglycerols (DAG) and triacylglycerols (TAG) [19]. Total intra- and extracellular FFA, DAG and TAG contents were assessed as the sum of particular fatty acid species (14:0, 16:0, 16:1, 17:0, 18:0, 18:1n9c, 18:2n6c, 20:0, 18:3n3, 22:0, 20:4n6, 24:0, 20:5n3, 24:1, 22:6n3) of the estimated fractions and expressed in nanomoles per concentration of protein in each sample.

### Data analysis

The data are expressed as mean values ± SD based on six independent determinations. Statistical difference between groups was tested two-way analysis of variance (ANOVA), using Statistica 10 (StatSoft, Krakow, Poland). The results were considered to be statistically significant at *P* ≤ 0.05.

## Results

### Effects of HepG2 cell incubation with PA and/or ENL on the intracellular FFA, DAG and TAG fraction contents

**Fig. 1:**
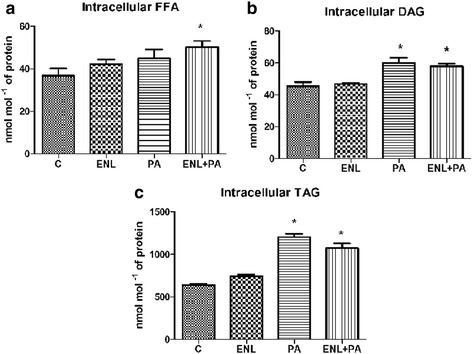
Intracellular content of free fatty acids (**a**, diacylglycerols (**b**) and triacylglycerols (**c**) in HepG2 cells. The cells were incubated with enterolactone (50 μM) alone or combined with palmitic acid (0.5 mM) for 16 h as it was described in details in Materials and Methods section. Total lipid content was measured by GLC method. The data are expressed as the mean ± S.D. . * P < 0.05 significant difference vs control group

The exposure of HepG2 cells to PA alone as well as PA combined with ENL induced a considerable intracellular DAG **(PA: +32.1%, ENL + PA: +27.6%;** **P < 0.05)** and TAG accumulation **(PA: +88.3%, ENL + PA: +68.2%;** **P < 0.05)** (Fig. 1b, c)**.** Moreover, we revealed that the intracellular FFA concentration in HepG2 cells was significantly elevated only after combined treatment (PA and ENL) as compared to the control group **(ENL + PA: +36.8%;** **P < 0.05**) (Fig. 1a).

### Effects of HepG2 cells incubation with PA and/or ENL on the extracellular FFA, DAG and TAG fraction contents

**Fig. 2:**
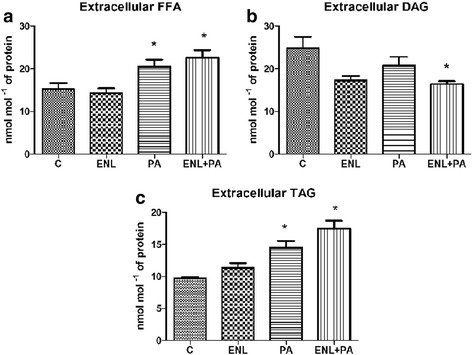
Extracellular content of free fatty acids (**a**), diacylglycerols (**b**) and triacylglycerols (**c**) in HepG2 cells. The cells were incubated with enterolactone (50 μM) alone or combined with palmitic acid (0.5 mM) for 16 h as it was described in details Materials and Methods section. Total lipid content was measured by GLC method. The data are expressed as the mean ± S.D. . * P < 0.05 significant difference vs control group

The incubation of HepG2 cells with PA alone or together with ENL significantly elevated the secretion of FFA into the media as compared with the control group **(PA: +34.8%, ENL + PA: +48.6%;** ***P*** **< 0.05)** (Fig. 2a). Unexpectedly, we also noticed that DAG concentration in postincubation medium was markedly decreased only in group exposed to ENL combined with PA in comparison to the control group **(ENL + PA: -33.8%;** ***P*** **< 0.05)** (Fig. 2b)**.** Similarly to FFA, we revealed a considerable accumulation of extracellular triacylgricerols in the media after PA alone as well as combined with ENL treatment **(PA: +41.9%, ENL + PA: +70.5%,** ***P*** **< 0.05)** (Fig. 2c)**.** Importantly, ENL alone did not affect FFA and TAG concentrations while its combination with palmitate resulted in a visible additive effect.

### Effects of HepG2 cells incubation with PA and/or ENL on total expression of proteins involved in extra- and intracellular lipid transport

**Fig. 3:**
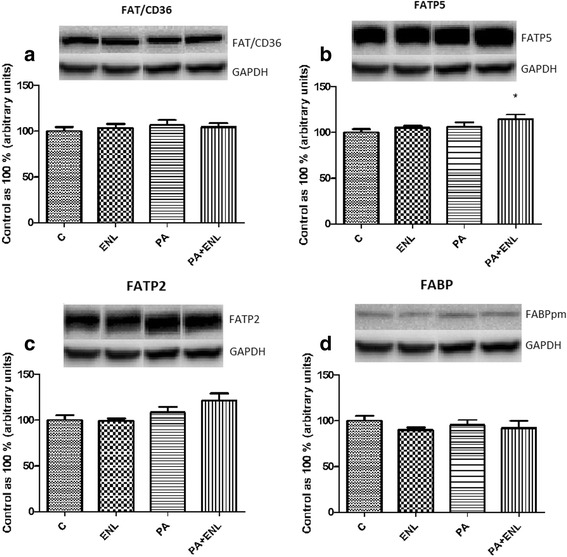
The expression of proteins involved in plasmalemmal lipid transport i.e. FAT/CD36 (**a**), FATP5 (**b**), FATP2 (**c**), FABPpm (**d**). The cells were incubated with enterolactone (50 μM) alone or combined with palmitic acid (0.5 mM) for 16 h as it was described in detail in see ‘Materials and Methods’ section. The protein expression in HepG2 cells was measured using Western blot method. The data are expressed as the mean ± S.D.* P < 0.05 significant difference vs control group

**Fig. 4:**
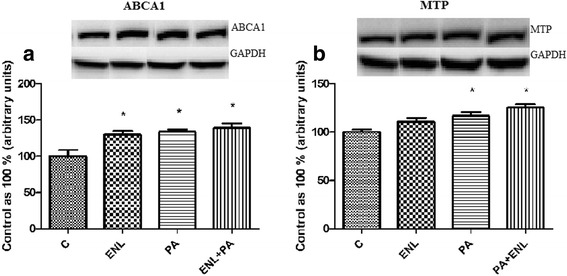
The expression of proteins involved in extracellular lipid transport i.e. ABCA1 (**a**), MTP (**b**). The cells were incubated with enterolactone (50 μM) alone or combined with palmitic acid (0.5 mM) for 16 h as it was described in details Materials and Methods section. The protein expression in HepG2 cells was measured using Western blot method. The data are expressed as the mean ± S.D. . * P < 0.05 significant difference vs control group

Among all the examined proteins involved in fatty acid uptake (FAT/CD36, FATP2, FATP5, FABPpm), we detected a significant increase only in FATP5 expression in PA-treated group exposed to enterolactone **(ENL + PA: +14.6%,** ***P*** **< 0.05)** (Fig. 3). Furthermore, in the case of extracellular lipid transporters there was a considerable rise in ABCA1 expression **(ENL: +29.6%,, PA: +33.6%, ENL + PA: +38.5%,** ***P*** **< 0.05)** (Fig. 4a) in all the examined groups compared to the control group, whereas MTP expression increased markedly only in the group incubated with PA alone or combined with ENL **(PA: +16.6%, ENL + PA: +25.3%**, ***P*** **< 0.05)** (Fig. 4b).

### Effects of HepG2 cells incubation with PA and/or ENL on total expression of cytosolic fatty acid transporters

**Fig. 5:**
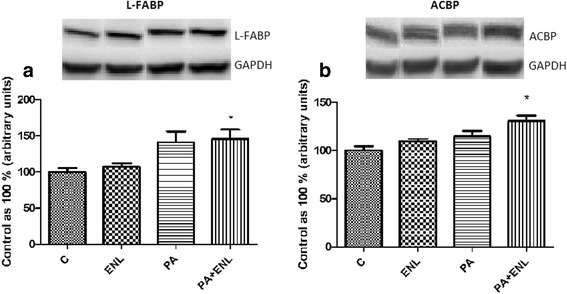
The expression of cytosolic fatty acids transporters i.e. L-FABP (**a**), ACBP (**b**). The cells were incubated with enterolactone (50 μM) alone or combined with palmitic acid (0.5 mM) for 16 h as it was described in details in Materials and Methods section. The protein expression in HepG2 cells was measured using Western blot method. The data are expressed as the mean ± S.D. * P < 0.05 significant difference vs control group

Total expression of L-FABP was significantly elevated in HepG2 cells exposed to PA alone as well as PA with ENL in comparison to the control group **(PA: +40.8%, ENL + PA: +45.7%,** ***P*** **< 0.05)** (Fig. 5a). On the other hand, the expression of ACBP was increased only after simultaneous treatment with both palmitic acid and enterolactone (**ENL + PA: +30.4%**, ***P*** **< 0.05)** (Fig. 5b).

### Effects of HepG2 cells incubation with PA and/or ENL on total expression of proteins involved in lipogenesis, lipid metabolism and oxidation pathway

**Fig. 6:**
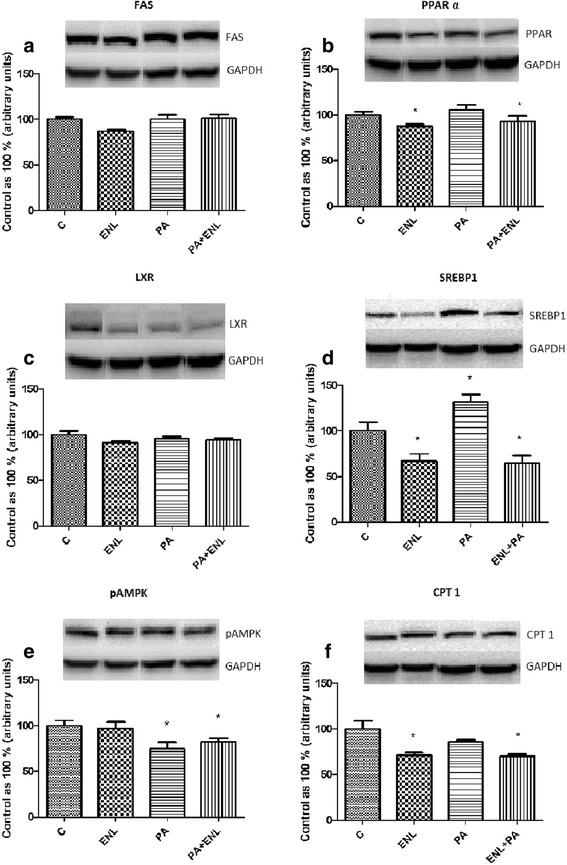
The expression of proteins involved in lipogenesis i.e. FAS (**a**), lipid metabolism i.e. PPARα (**b**), LXR (**c**), SREBP1c (**d**), pAMPK (**e**) and oxidation pathway i.e. CPT 1 (**f**). The cells were incubated with enterolactone (50 μM) alone or combined with palmitic acid (0.5 mM) for 16 h as it was described in details in Materials and Methods section. The protein expression in HepG2 cells was measured using Western blot method. The data are expressed as the mean ± S.D. * P < 0.05 significant difference vs control group

In our study we demonstrated that enterolactone has no influence on FAS and LXR (Fig. 6a, c) expression in HepG2 cells. Furthermore, ENL alone or combined with PA decreased significantly PPARα **(ENL: -14.8%, PA + ENL: -14.6%,** ***P*** **< 0.05)** (Fig. 6b) and slightly reduced pAMPK **(PA: -21.9%, ENL+ PA: -14.5%**, *P* **< 0.05)** (Fig. 6e) expression in comparison to the control group. Moreover, we observed a substantial decline in CPT 1 expression caused by incubation with both PA and PA combined with ENL **(PA: -26.3%, ENL + PA: -30.6%**, *P* **< 0.05)** (Fig. 6f). Interestingly, ENL both alone or combined with PA decreased expression of sterol regulatory element-binding protein (SREBP1c) compared to the control group counteracting PA related activation **(ENL: -23.1%, PA: +45%, PA + ENL: -28.7%**, *P* **< 0.05)** (Fig. 6d).

## Discussion

One of the main pathological factors of NAFLD development is an increased intracellular TAG content which can be caused by high fat diet administration [4]. It is well known that this change can also be a result of disturbed fatty acids transmembrane transport as well as impaired lipid metabolism in hepatocytes [20]. Both of these processes are possible targets for NAFLD treatment and can be affected by ENL, a flaxseed derived polyphenol [6]. We conducted our experiments on HepG2 cells, being immortalized human liver cell line that still preserve a number of normal liver functions (lipid accumulation, glycogen storage, urea synthesis, phase I and phase II drug metabolizing activities) to assess the influence of ENL treatment in different metabolic conditions (overnutrition) on fatty acid transporters and lipid metabolism. As our study design limitation is not to reflect normally observed in human plasma ENL concentration, based on pilot studies, we considered it to be the most effective [21, 22]. Future studies should be directed at investigating physiological ENL concentration effects or finding new ENL metabolites that are less toxic with the same positive properties. In hepatocytes the transport of fatty acids between extracellular fluid (ECF) and intracellular fluid (ICF) is a result of a few co-existing processes [20, 23]. Within them we might distinguish fatty acids uptake facilitated by protein transporters (FAT/CD36, FATP2, FATP5, FABPpm), lipid efflux conducted by ABCA1 and MTP, and concentration gradient-driven diffusion depicted by ACBP, and L-FABP [24, 25]. At present, there are no other investigations reporting the influence of ENL on fatty acid transporters. In our research, ENL supplementation in lipid overload condition did not have any significant influence on FAT/CD 36, FATP2 nor FABPpm transport proteins, a set of transport proteins regulating cellular long chain fatty acids (LCFA) uptake [24, 25]. However, it slightly increased the expression of FATP-5. Probably low expression of the transport proteins in HepG2 cells in comparison to primary hepatocytes in general may be a reason why the family of FATP transporters does not play a main role in fatty acid transport in this type of cells [7]. This may suggest that in HepG2 transmembrane transport of fatty acids is mainly a result of high intracellular diffusion gradient via L-FABP rather than mediated by protein transporters because changes observed in their expression were quite small. We only observed significant changes in FATP-5 expression which, at this condition, may be sufficient enough to increase FA influx. Moreover, it appears that this effect may be associated with observed simultaneously decreased expression of pAMPK and PPARα, which are activators of lipids uptake via fatty acid transporters such as: FAT/CD36, FATP-2 or FATP-5 [26, 27]. What is more, ENL supplementation stimulated ACBP and L-FABP mediated long and medium-chain fatty acids (LCFA, MCFA) binding and their transport between organelles and outside the cells, protecting hepatocytes from oxidative damage and cytotoxicity of free fatty acids [28]. However, significantly increased L-FABP expression is not correlated with PPARα changes what is surprising since peroxisome proliferator acivated receptor stimulates its activity. We suspect that it may be other than PPAR alpha activating mechanisms, especially in HepG2 cells, or excessive lipid availability directly increased the expression of this transporter. The second type of transport (diffusion) was correlated with increased expression of ABCA1 and MTP, suggesting ENL related intensification of fatty acid efflux [29]. ABCA1 which is a transmembrane LXR controlled regulatory protein, together with MTP, another transmembrane extracellular transporter, are crucial for cholesterol and phospholipids efflux and thus necessary for synthesis of HDL, and ApoB lipoprotein [30, 31]. Our study showed that ENL combined with PA increased the efflux of FFA and TAG to the media, decreasing extracellular DAG concentration at the same time. Both ABCA1 and MTP regulate ECF lipid profile, ejecting excess of fatty acids outside the hepatocytes [23, 30]. Nevertheless, a bit different effects of ENL were observed in an animal model. In line with this study also other authors shown that SECO and its metabolites such as enterodiol (END) and ENL in vivo decreased expression of SREBP1c [32]. However, above substances have also increased the expression of PPARα, acyl-CoA oxidase (ACOX) and CPT 1 genes what have not been observed in our investigation [32]. Furthermore, supplementation of above-mentioned polyphenols resulted in a decreased body weight gain via increased secretion of leptin what might explain their beneficial role in liver steatosis and obesity in animals [32, 33]. Nevertheless, in our experiment no significant changes have been observed in lipid fractions concentration as a result of only ENL supplementation. On the other hand, when we consider intracellular lipid content, ENL mixed with PA significantly increased FFA concentration, similarly to extracellular fluid (ECF). Moreover, ENL combined with PA slightly reduced the PA evoked augmentation of DAG and TAG compared to ENL alone. Furthermore, simultaneous elevation of TAG concentration in examined media may suggest that the addition of ENL promotes its transport outside the hepatocytes, which might be beneficial, protecting the liver from lipid overload. What is more, ENL significantly reduced carnitine palmitoyltransferase 1 (CPT 1) expression, which is responsible for mitochondrial fatty acid transport, and thus lipid oxidation in hepatocytes [34]. In contrast to our results, Tominaga in vivo study showed no effect of low ENL dosage on CPT 1 genes expression and its stimulation in high ones [32]. We can suspect that this discrepancy was caused by different doses of ENL, which in the case of our research was high enough to trigger changes in protein expression. Nevertheless, when we consider our experiment, the reduction in CPT 1 expression was correlated with a decreased expression of pAMPK, a protein junction point between lipid oxidation and de novo synthesis pathway [35, 36]. Decrease of AMPK stimulated TAG and FFA synthesis in PA and ENL + PA groups and inhibited lipid oxidation. These findings suggest that, inhibited fatty acids delivery to mitochondria and thus impaired oxidation resulted in an excessive lipid accumulation [37]. Furthermore, ENL both alone and combined with PA significantly reduced PPARα expression contributing to inhibited fatty acids oxidation, decreased fatty acids uptake, and probably declined CPT 1 expression. It is widely known that PPARα regulates not only lipids but also carbohydrates metabolism in hepatocytes [38]. Studies conducted by many researchers have shown that lower PPARα expression favors hepatic glucose production and is also correlated with insulin resistance, causing deterioration of NAFLD [39–41]. However, Pan et al. did not notice significant changes in insulin sensivity after supplementation with flaxseed-derived lignans in type 2 diabetic patients [42]. As far as lipid metabolism is concerned, our observations also indicate that ENL would likely have an influence on fatty acid de novo synthesis pathway by decreasing the expression of SREBP1c what might suggest antisteatotic effect of ENL in the incubated cells. Surprisingly, this result did not correlate with significant changes neither in liver X receptor (LXR), nor FAS expression what have been frequently observed in NAFLD patients [38, 43]. We can suspect that this discrepancy may be either a result of selective influence of ENL on FAS and SREBP1c or increased transmembrane transport was sufficient enough to cause TAG, DAG accumulation, especially when FA oxidation remained not changed. According to our results, ENL had a opposite effect on lipid transport and metabolism in comparison to its main source product SECO and other commonly examined lignans such as: resveratrol (RSV), kukoamine A or quercetin (QUE) [35, 44, 45]. We cannot exclude the possibility that ENL will show similar properties to Schisandrin B (Sch B), an active dibenzooctadiene lignan which presents an antihyperlipidemic and hepatoprotective effects only when is supplemented for a long time in a low dosage [46, 47]. On the other hand, when Sch B was applied in a single high dose bolus it increased serum lipid and cholesterol level in NAFLD mice [47]. Analogical effect was observed in our experiment where ENL supplementation resulted in increased fatty acid efflux from lipid overloaded hepatocytes which lipid storage capacity was exceeded and the cell was defending against lipotoxicity via increased ABCA1 and MTP expression. It is possible that in lower concentration and during prolonged exposure, similarly to Sch B, ENL will express antihyperlipidemic and hepatoprotective effects increasing lipids uptake and their further oxidation. That is why, ENL should be tested in various combinations of dose/time exposure to broadly verify its effects on lipid transporters and lipid metabolism.

## Conclusions

The findings of our study demonstrate that ENL had minor and opposite effect on liver steatosis in comparison to other polyphenols which suggests that it may be an less active metabolite. ENL did not affect significantly accumulation of FFA, DAG and TAG. Furthermore, it had no influence on fatty acid transport proteins expression, yet it promoted lipids extracellular efflux. Moreover, ENL inhibited ß-oxydation and intracellular lipid metabolism via PPARα what may contribute to the progression of NAFLD. Nevertheless, it selectively inhibited SREBP1c and slightly reduced PA induced inhibition of pAMPK. However, further investigations are still needed for complex verification of metabolic effects of ENL and to assess its potential usage in clinical treatment of NAFLD.

## Abbreviations

ABCA1: ATP-binding cassette transporter 1
ACBP: Acyl binding protein
ACOX: acyl-CoA oxidase
ATCC: American Type Culture Collection
BSA: Fatty acid-free bovine serum albumin
CPT 1: Carnitine palmitoyl transferase 1
DAG: Diacylglycerols
DMEM: Dulbecco Modified Eagle Medium
ECF: Extracellular fluid
ENL: Enterolactone
FABPpm: Fatty acid binding protein
FAT/CD36: Fatty acid translocase
FATP: Fatty acid transport protein
FBS: Fetal bovine serum
FFA: Free fatty acids
GAPDH: Glyceraldehyde 3-phosphate dehydrogenase
GLC: Gas liquid chromatography
HDL: High-density lipoprotein
HRP: Horseradish peroxidase
ICF: Intracellular fluid
LCFA: Long chain fatty acid
L-FABP: Liver-type fatty acid binding protein
LXR: Liver X receptor
MCFA: Medium chain fatty acid
MTP: Mitochondrial trifunctional protein
NAFLD: Non-alcoholic fatty liver disease
NASH: Non-alcoholic steatohepatitis
PA: Palmitic acid
pAMPK: phosphorylated 5′ AMP-activated protein kinase
PPARα: Peroxisome proliferator-activated receptor α
QUE: Quercetin
RSV: Resveratrol
Sch B: Schisandrin B
SECO: Secoisolariciresinol
SREBP1c: Sterol regulatory element-binding protein 1c
TAG: Triacylglycerols
TLC: Thin-layer chromatography
TNB: Thick needle biopsy
